# Temperature and aridity determine body size conformity to Bergmann’s rule independent of latitudinal differences in a tropical environment

**DOI:** 10.1007/s10336-018-1574-8

**Published:** 2018-06-27

**Authors:** Chima J. Nwaogu, B. Irene Tieleman, Kwanye Bitrus, Will Cresswell

**Affiliations:** 1 Groningen Institute for Evolutionary Life Sciences, University of Groningen, P.O. Box 11103, 9700 CC Groningen, The Netherlands; 2 School of Biology, University of St Andrews, Harold Mitchell Building, St Andrews, Fife KY16 9TH UK; 3 A. P. Leventis Ornithological Research Institute, Jos, Nigeria; 4 Department of Evolutionary Zoology and Human Biology, University of Debrecen, Egyetem tèr 1, Debrecen, 4032 Hungary

**Keywords:** Latitude, Thermoregulation, Global warming, Birds, Temperature variation, Bioclimatic variable

## Abstract

Bergmann’s rule, defined as the tendency for endotherms to be larger in colder environments, is a biophysical generalization of body size variation that is frequently tested along latitudinal gradients, even though latitude is only a proxy for temperature variation. We test whether variation in temperature and aridity determine avian body size conformity to Bergmann’s rule independent of latitude differences, using the ubiquitous Common Bulbul *Pycnonotus barbatus*, along a West African environmental gradient. We trapped 538 birds in 22 locations between latitudes 6 and 13°N in Nigeria, and estimated average body surface area to mass ratio per location. We then modelled body surface to mass ratio using general linear models, with latitude, altitude and one of 19 bioclimatic variables extracted from http://www.worldclim.org/bioclim as predictors. We sequentially dropped latitude and altitude from each model to obtain the *R*^2^ of the resultant models. Finally, we compared the *R*^2^ of univariate models, where bioclimatic variables predicted body surface area to mass ratio significantly (14 out of 19), to multivariate models including latitude, altitude and a bioclimatic variable, using the Wilcoxon matched pairs test. We found that multivariate models did not perform better than univariate models with only bioclimatic variables. Six temperature and eight precipitation variables significantly predicted variation in body surface area to mass ratio between locations; in fact, 50% (seven out of 14) of these better explained variation in body surface area to mass ratio than the multivariate models. Birds showed a larger body surface area relative to body mass ratio in hotter environments independent of latitude or altitude, which conforms to Bergmann’s rule. Yet, a combination of morphometric analyses and controlled temperature-exposure experiments is required to prove the proposed relationship between relative body surface area and thermoregulation in endotherms.

**Electronic supplementary material:**

The online version of this article (10.1007/s10336-018-1574-8) contains supplementary material, which is available to authorized users.

## Zusammenfassung

**Temperatur und Trockenheit bestimmen in Einklang mit der Bergmannschen Regel in tropischer Umgebung die Körpergröße, unabhängig von der geographischen Breite**

Die Bergmannsche Regel, nach der Warmblüter dazu tendieren, in kalter Umgebung größer zu werden, ist eine biophysikalische Verallgemeinerung, die immer wieder an unterschiedlichen Breitengraden getestet wird, obwohl die geographische Breite eigentlich stellvertretend für einen Temperaturgradienten steht. Wir untersuchten entlang eines westafrikanischen Gradienten und mit dem dort überall vorkommenden Graubülbül (*Pycnonotus barbatus*), ob unabhängig von unterschiedlichen Breitengraden die Unterschiede in Umgebungstemperatur und Trockenheit die Körpergröße bestimmen und dabei der Bergmannschen Regel folgen würden. Wir fingen 538 Vögel an 22 Orten zwischen dem 6. und dem 13. Grad nördlicher Breite in Nigeria und bestimmten für jeden einzelnen Ort das Verhältnis von mittlerer Körperoberfläche zur Körpermasse. Anschließend stellten wir Allgemeine Lineare Modelle für das Verhältnis von Körperoberfläche zur -masse auf, unter Berücksichtigung der geographischen Breite, der Höhe und einer von 19 Bioklimatologie-Variablen (entnommen bei: http://www.worldclim.org/bioclim) als Prädiktoren. Wir eliminierten der Reihe nach die geographische Breite aus jeder Modellrechnung, um zu R-Quadrat-Werten der resultierenden Modelle zu gelangen. Schließlich verglichen wir mit dem Wilson Matched-Pair-Test die R-Quadrat-Werte der univariaten Modelle, in denen die bioklimatologischen Variablen das Verhältnis von Körperoberfläche signifikant (14 von 19) zur Masse vorhersagten, mit den multivariaten Modellen, die die geographische Breite, die Höhe und bioklimatische Variablen beinhalteten. Wir stellten fest, dass multivariate Modelle nicht besser waren als univariate nur mit bioklimatologischen Variablen. Sechs Temperatur- und acht Niederschlags-Variablen reichten, um signifikante Vorhersagen zu den je nach Ort unterschiedlichen Relationen von Körperoberfläche zu -masse zu machen. Fünfzig Prozent (7 der 14) gaben bessere Erklärungen für die unterschiedlichen Körperoberfläche-zu-Masse-Verhältnisse als die multivariaten Modelle. Unter heißeren Umgebungsbedingungen hatten die Vögel relativ zur Körpermasse eine größere Körperoberfläche, unabhängig von der geographischen Breite oder Höhe, und das ist konform mit der Bergmannschen Regel. Aber es ist noch eine Kombination von morphometrischen Analysen mit Experimenten notwendig, in denen Tiere kontrollierten Temperaturen ausgesetzt werden, um den hier vorgeschlagenen Zusammenhang zwischen relativer Körperoberfläche und Thermoregulation bei Warmblütern beweisen zu können.

## Introduction

Bergmann’s rule, defined as the tendency for endotherms to be larger in colder environments, is a well-known biophysical generalization for thermoregulation along environmental gradients (Salewski and Watt 2017). The rule applies to structural adaptation for thermoregulation in endotherms as observed in other animals (Porter and Kearney 2009; Greenberg et al. 2012; Glanville et al. 2012) rather than simple spatial body size variation, and this distinction is important. Bergmann’s rule is based on adaptation to local temperature differences independent of variation across space, although such variation, usually with latitude, is used to test the rule (Gardner et al. 2011).

The usefulness of Bergmann’s rule has received renewed attention for assessing the impact of global warming or presenting additional evidence for climate change via variation in animal body size over time (Gardner et al. 2011). But the validity of the rule is largely debated (Scholander 1955; Mayr 1956; Geist 1987; McNab 1971, 2010; Yom-Tov and Geffen 2011) due to inconsistent body size patterns along latitudinal gradients. It seems likely that the exploration of absolute body size patterns (Graves 1991; Meiri and Dayan 2003; Ashton 2002) rather than relative body surface area in relation to thermoregulation (Salewski and Watt 2017) and the use of latitude as surrogate for temperature variation (Meiri and Dayan 2003) are responsible for some of the inconsistencies observed. Apart from Bergmann’s rule, there are other ecogeographical hypotheses relating animal body size to environmental conditions, and although these are not mutually exclusive from Bergmann’s rule, most address absolute measurements of body parts while Bergmann’s rule is based on relative body surface area. Allen’s rule postulates that animals should have longer body extremities in warmer environments (Allen 1877), while Niles (1973) reports larger Horned Larks *Eremophila alpestris* in areas of higher environmental productivity and Mayr (1957) shows size increases with elevation. Bergmann’s rule, on the other hand, specifically postulates structural adaptation of the entire animal body to environmental temperature, based on a biophysical principle that more heat is lost to the external environment as body surface area increases relative to volume (Harley et al. 2009; Salewski and Watt 2017). In principle, species that are structurally longer and less compact in warmer environments, but relatively plump and compact in cooler ones, consistent with Allen’s rule, may conform to Bergmann’s rule as well, because elongated body forms should have relatively larger surface area to volume ratio. Furthermore, variation in the size of body reserves or structural size due to environmental productivity (Madsen and Shine 2000) may also cause variability in surface area to volume ratio. In essence, body surface area to volume ratio should decrease as an animal’s environment becomes colder, so that less of its produced heat is lost to its environment. Consequently, when heat loss is required to maintain relatively constant body temperatures in hot environments, we might expect a relatively larger surface area to volume ratio. Bergmann’s rule should be interpreted based on two measures of body size: the ratio of the area to a cubic measure of body size.

Latitude encompasses many potentially confounding effects (Meiri and Dayan 2003), such as altitude, aridity, vegetation structure and food availability, and all these may affect temperature and body size differently. In birds, measures of body mass combine body size and body reserves (Gosler et al. 1998), and body reserves vary rapidly due to predation or starvation risk (Lima 1986), so mass alone cannot be used as a measure of body size (Piersma and Davidson 1991; Cresswell 2009). Physical and biological processes which affect body size or reserves such as food supply during growth (Madsen and Shine 2000), starvation/predation risk (Cresswell 1998), breeding (Nwaogu et al. 2017) and migration (Åkesson et al. 1992; Nwaogu and Cresswell 2015) may vary with latitude and confound temperature effects. Bergmann’s rule is therefore interpreted best based on the local environmental conditions of living animals and not their latitudes, and with live body size measures within a single resident species, so as to eliminate species-specific thermoregulatory adaptations (Scholander 1955).

In this study we test the relationship between body surface area to mass ratio and environmental factors in a West African tropical environment to find out whether environmental temperature variation determines body size conformity to Bergmann’s rule independent of latitude. We estimated body surface area as the square of wing length and used body mass as a proxy for volume in the Common Bulbul *Pycnonotus barbatus,* a ubiquitous resident tropical songbird. We correlate surface area to mass ratio with 19 climatic variables that explain local environmental conditions. We predict that populations of Common Bulbuls in hotter and more arid environments will have larger body surface area to mass ratios independent of latitude. However, since body mass can be highly variable and may result in variation in body surface area to mass ratio even without a variation in body size across latitude, we also modelled variation in body mass and wing length on their own. We provide evidence that wing length, but not body mass, increases significantly northwards with increasing temperature and aridity, hence patterns of body surface to mass ratio can reliably be interpreted as conforming to Bergmann’s rule.

## Method

### Study area

This study was carried out within Nigeria; birds were mist-netted between latitude 6 and 13°N, and 49–1716 m a.s.l. West Africa is bounded to the north by the Sahara desert and to the south and west by the Atlantic Ocean. This feature creates a gradient of temperature and aridity from the dry edge of the Sahara Desert in the north to the wet coastal areas of the Atlantic Ocean in the south of Nigeria, and this contrasts with the pattern of the larger scale global gradient of decreasing temperature from the equator to the North Pole. There is also a large variation in altitude and vegetation structure between locations, and this is not entirely consistent with latitudinal differences. Precipitation is seasonal in West Africa but humidity and environmental temperature do not follow similar patterns across the year. The increase in rainfall is unimodal but temperature variation is bimodal because of low temperatures during the dusty Harmattan weather in the dry season and at the peak of the wet season. Differences in humidity due to interactions between temperature and rainfall may affect the water balance between organisms and their environment and thus, thermoregulation, so we also consider the effect of precipitation variables on body surface area to mass ratio. There is usually a single period of rainfall, hence one of drought annually, but the extent of the wet season varies between locations. The wet season is later and shorter in northern latitudes, which are more arid compared to southerly ones (or higher altitude locations), and this combines with altitudinal differences and vegetation structure to determine local climates which are largely independent of latitude variation in space.

### Study species

The Common Bulbul is widespread and resident throughout Africa. Common Bulbuls are sexually monomorphic, usually 9–11 cm in body length and weigh 25–50 g. Adult birds feed on fruits, insects, nectar and seeds. Fruits are generally available to Bulbuls year-round but from different plants that vary in fruiting phenology.

### Determination of variables

We obtained body size measurements from a total of 538 Common Bulbuls from 22 locations in Nigeria. We trapped 308 of 538 Common Bulbuls from 15 locations between 17 January and 8 April 2017, while data for an additional 230 birds from seven locations were obtained from our past ringing records archived in the A. P. Leventis Ornithological Research Institute ringing database collected between 2001 and 2016 (Cox et al. 2011). All birds were caught using mist nets from 0600 to 1030 hours. For each trapped bird, we recorded wing length (± 1 mm), pectoral muscle score, fat score and body mass (± 0.1 g; Ohaus Scout) (Svensson 1992; Redfern and Clark 2001). Tarsus length was also measured for birds trapped in 2017. We estimated average body surface area to mass ratio by dividing the square of wing length by body mass for each individual bird. We extracted 19 local bioclimatic variables for each capture location (Table 1), including 11 temperature and eight precipitation variables from http://www.worldclim.org/bioclim, using the maptools and raster packages in R. We relied on wing length and body mass measurements as proxies of body size, because both are more often accurately obtained by ringers (Gosler et al. 1998), although their accuracy as a proxy for body size may vary among species (Rising and Somers 1989; Senar and Pascual 1997). Our method is easily repeatable using records from avian ringing databases for the same species in different locations. However, to validate the reliability of squared wing length as a proxy for body surface area, we correlated squared wing length, and the product of wing and tarsus lengths, since both are linear size measures of the same individual whose product gives an area measure similar to the square of wing length [*r* = 0.79, df = 289, *p* < 0.0001, see Fig. 2]. We used the square of wing length for all our analyses because we only have tarsus length measurements for 15 out of 22 locations. This should be consistent with Bergmann’s rule of body surface area to volume ratio [using measures of wingspan as proxy for body size (Salewski and Watt 2017)], because wing length is linear and body mass is a similar cubic measure to volume.

**Table 1:** Adjusted *R*^2^ of general linear models explaining body surface area to mass ratio of Common Bulbuls *Pycnonotus barbatus* across environmental conditions in West Africa

	*R*^2^ of univariate model				Δ*R*^2^^a^		
	Climatic variable	Latitude	Altitude	Full model^b^	Climatic variable	Latitude	Altitude
Annual mean temperature	− 0.01	0.48	− 0.05	0.47	− 0.01	− 0.15	0
Mean diurnal range [mean of monthly (max. temp—min. temp)]	*0.5*	0.48	− 0.05	*0.48*	− *0.02*	*0.03*	*0.01*
Isothermality	*0.63*	0.48	− 0.05	*0.6*	− *0.14*	*0.02*	*0.01*
Temperature seasonality (SD × 100)	*0.5*	0.48	− 0.05	*0.46*	*0*	*0.02*	*0.03*
Max. temperature of warmest month	*0.33*	0.48	− 0.05	*0.52*	− *0.06*	*0.03*	− *0.03*
Min. temperature of coldest month	*0.25*	0.48	− 0.05	*0.51*	− *0.05*	*0.02*	− *0.05*
Temperature: annual range	*0.58*	0.48	− 0.05	*0.54*	− *0.08*	*0.02*	*0.02*
Mean temperature of wettest quarter	0.009	0.48	− 0.05	0.44	0.02	− 0.12	0.03
Mean temperature of driest quarter	− 0.03	0.48	− 0.05	0.43	0.03	− 0.22	0.03
Mean temperature of warmest quarter	0.13	0.48	− 0.05	0.52	− 0.06	0.02	− 0.04
Mean temperature of coldest quarter	− 0.05	0.48	− 0.05	0.45	0.01	− 0.55	0.02
Annual precipitation	*0.55*	0.48	− 0.05	*0.51*	− *0.05*	*0.02*	*0.03*
Precipitation of the wettest month	*0.22*	0.48	− 0.05	*0.44*	*0.02*	− *0.23*	*0.03*
Precipitation of the driest month	*0.49*	0.48	− 0.05	*0.51*	− *0.05*	− *0.05*	*0.02*
Precipitation seasonality (coefficient of variation)	*0.57*	0.48	− 0.05	*0.53*	− *0.07*	*0.02*	*0.02*
Precipitation of the wettest quarter	*0.26*	0.48	− 0.05	*0.44*	*0.02*	− *0.19*	*0.03*
Precipitation of driest quarter	*0.56*	0.48	− 0.05	*0.55*	− *0.09*	− *0.01*	*0.01*
Precipitation of warmest quarter	*0.51*	0.48	− 0.05	*0.57*	− *0.11*	− *0.06*	*0.01*
Precipitation of coldest quarter	*0.37*	0.48	− 0.05	*0.44*	*0.02*	− *0.07*	*0.03*

Models where a bioclimatic variable made a significant contribution to explaining the body surface area to mass ratio are indicated in* italic*

*max.* Maximum, *min. * minimum

^a^Change in adjusted *R*^2^ is the difference that results from dropping the variable in a column from the full model with all three variables

^b^Full model includes latitude, altitude and one bioclimatic variable as predictors

### Statistical analyses

We built a general linear model (GLM) to predict average body surface area to mass ratio for each of the 22 capture locations where we trapped birds. We included pectoral muscle, subcutaneous fat scores and moult stage of birds in the GLM, but they did not improve model fit, hence they were dropped from the final model. Presence of brood patch (as a proxy for breeding status) explained 3% additional variation in body surface to mass ratio, but because only female birds carry brood patches, we could not control for breeding status when calculating predicted body surface area to mass ratio per location. Ignoring breeding status was unlikely to affect our conclusions because breeding in the Common Bulbul does not follow a consistent pattern along an environmental gradient (unpublished data; see also Fig. 2). The final model with which we estimated body surface area to mass ratio included only capture location as a predictor variable (*r* = 0.36, df = 516, *p* < 0.0001). We then obtained predicted body surface area to mass ratio for each location using the predict function in R. Subsequently, we built a GLM to model predicted body surface area to mass ratio per location by latitude, altitude and one of 19 bioclimatic variables (Table 1). For each model, we sequentially dropped latitude and altitude to obtain *R*^2^ of resultant models (each including only a single local climatic variable).

We repeated the same analyses (as we did with body surface area to mass ratio) using body mass and wing length on their own because both body mass and size may vary independently due to factors unrelated to thermoregulation, thus, confound observations. Body mass should be higher in wetter and cooler environments where birds may breed earlier and thus, carry extra body reserves due to interrupted foraging (Macleod and Gosler 2006; Nwaogu et al. 2017). Also wing length should be shorter in more arid environments if net primary productivity determines overall body size (Hilderbrand et al. 1999; Madsen and Shine 2000), or relatively longer if Allen’s rule is valid (Allen 1877). Compared to body surface area to mass ratio, variation in body mass and wing length alone were less well explained by bioclimatic variables (Tables S1, S2), thus it seems likely that there were no significant confounding effects of breeding or food availability on the estimated body surface to mass ratio.

To test the predictive power of bioclimatic variables independent of latitude, we compared the adjusted *R*^2^ of univariate models where bioclimatic variables predicted body surface area to mass ratio significantly (14 out of 19; see Table 1) to multivariate models including latitude, altitude and a bioclimatic variable, using the Wilcoxon matched pairs test. This was to confirm whether univariate models with a bioclimatic variable alone generally explained variation in body surface to mass ratio without including latitude and altitude in models. All analyses were carried out in R version 3.4.1 (R Development Core Team 2018).

## Results

**Fig. 1:**
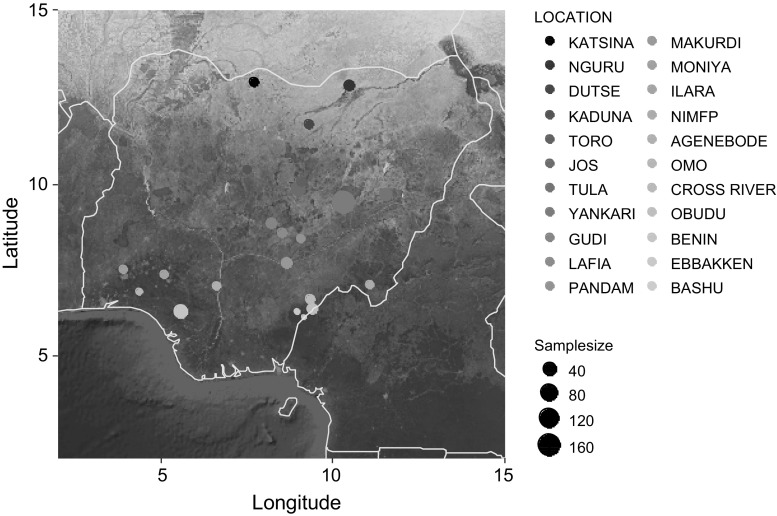
Map showing distribution of locations where body size measurements of Common Bulbuls *Pycnonotus barbatus* were obtained across West Africa. Sampling points are* shaded darker* with increasing latitude consistent with Figs. 2, 3 and 4

Body surface area to mass ratio of Common Bulbuls across different environments was determined by local bioclimatic variables independent of latitude (Table 1; Figs. 3, 4). Birds had larger body surface area to mass ratio in hotter, arid and more seasonal environments compared to colder, wetter and less seasonal ones in Nigeria independent of latitude (Figs. 1, 3, 4). Body surface area to mass ratio was predicted significantly by 14 of the 19 bioclimatic variables (see Table 1 for significant variables). Mean annual temperature, and temperatures of the wettest, driest, warmest and coldest quarters, did not explain much variation in body surface area to body mass ratio between locations (Table 1). Multivariate models including latitude and altitude as predictor variables were not better at explaining variation in body surface area to body mass ratio of Common Bulbuls compared to univariate models of each of the 14 significant local bioclimatic variables alone (*V* = 71.5, *p* = 0.12, median = 0.51 vs. 0.50, *n* = 14). Climatic variables alone explained between 0 and 63% of the variation in body surface area to mass ratio of Common Bulbuls across locations (Table 1). Latitude alone explained 48% while altitude alone explained 0% of the variation in body surface area to mass ratio of Common Bulbuls across locations (Table 1). Nine out of 14 (64%) significant univariate models for bioclimatic variables had a higher *R*^2^ than univariate models using latitude alone (Table 1). For the explorations of variation in wing length and body mass alone (Tables S1, S2), bioclimatic variables explained 0–45 and 0–26% variation in wing length and body mass, respectively. Latitude explained 30 and 13% variation in wing length and body mass, respectively, while altitude explained 11 and 3% of wing length and body mass respectively.

## Discussion

**Fig. 2:**
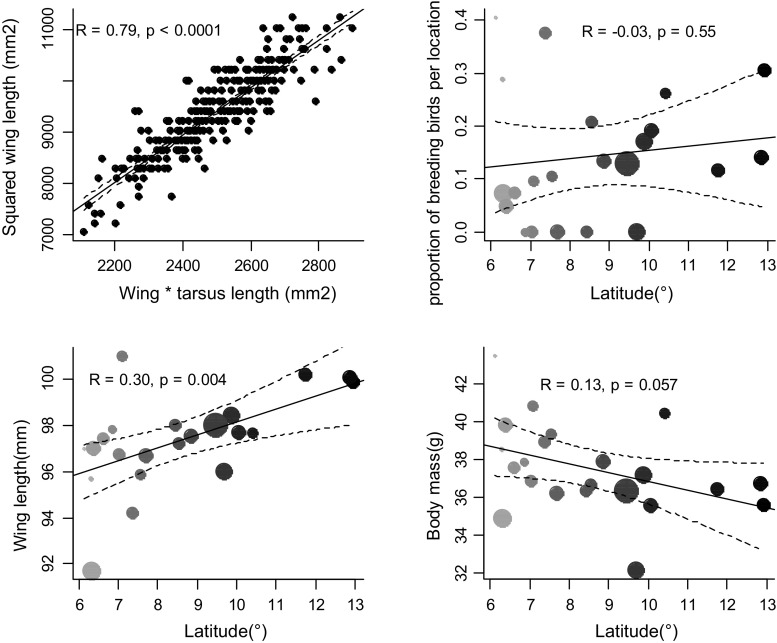
*Top left* Correlation between squared wing length and product of wing and tarsus lengths of 308 Common Bulbul *P. barbatus* trapped in 15 out of 22 study sites along the temperature gradient in Nigeria.* Top right* No correlation between log proportion of breeding birds per location and latitude.* Bottom left* Positive correlation between wing length and latitude suggests birds are significantly bigger at higher latitudes.* Bottom right* No correlation between body mass and latitude—note that higher latitude locations are more likely to be arid (see Fig. 1).* Grey shading* from* light* to* dark* is consistent with increasing latitude and* point sizes* indicate sample size (see Fig. 1)

**Fig. 3:**
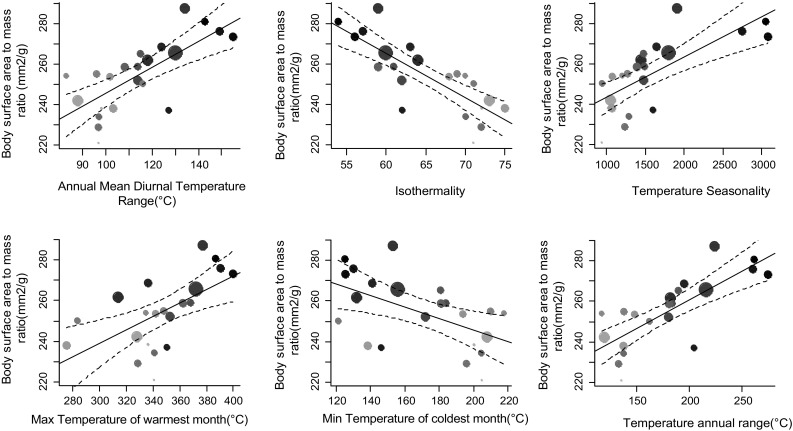
Correlations between body surface area to mass ratio of Common Bulbuls *P. barbatus* and six significant temperature variable predictors.* Grey scale* is ordered by increasing latitude to show independence of body surface to mass ratio and latitude.* Grey shading* from* light* to* dark* is consistent with increasing latitude, and* point sizes* indicate sample size (see Fig. 1)

**Fig. 4:**
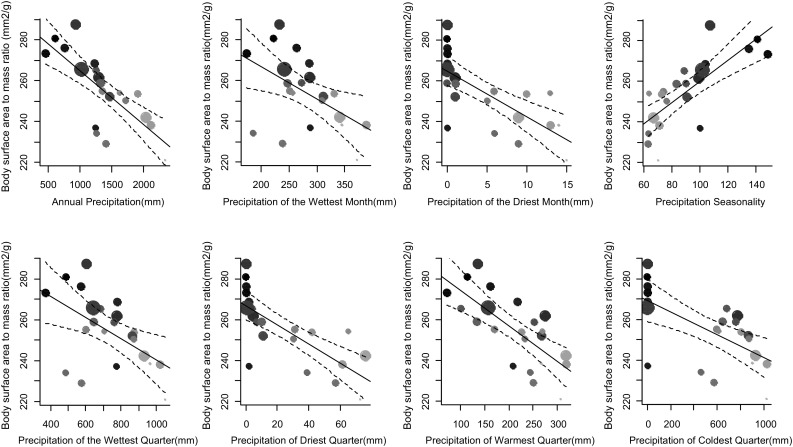
Correlations between body surface area to mass ratio of Common Bulbuls *P. barbatus* and eight significant precipitation variable predictors.* Grey scale* is ordered by increasing latitude to show independence of body surface to mass ratio and latitude.* Grey shading* from* light* to* dark* is consistent with increasing latitude and* point sizes* indicate sample size (see Fig. 1)

Our results suggest that the link between relative body surface area and temperature variation along environmental gradient is valid and possibly related to thermoregulation in the Common Bulbul after taking local environmental conditions (Figs. 3, 4, Table 1), absolute body size variation (Fig. 2, Tables S1, S2) and time of capture (Table S3) into account. We discuss these results that show that patterns of variation in body surface area to body mass ratio of a tropical songbird are consistent with Bergmann’s rule independent of latitude.

Temperature varies globally on a latitudinal scale, but using latitude as a proxy for temperature variation may be misleading if local factors override global patterns (Meiri et al. 2007; Bourgault et al. 2010). Our data confirm that latitudinal differences do not often capture the combined effect of local factors on environmental conditions (Bourgault et al. 2010), because bioclimatic variables were relatively better at explaining body surface area to mass ratio compared to latitude (Table 1). In this case, body surface area to mass ratio also correlated with latitude, because temperature and aridity vary from north to south with timing and duration of rainfall in contrast with the global pattern of increasing temperature towards the pole, but consistent with decreasing local temperatures towards the Atlantic (Fig. 1). This shows a strong association of body size variation and environmental temperature despite a reversal of the global latitudinal pattern.

Environmental temperature and aridity are closely linked (James 1970) and this may affect internal water balance (Tieleman and Williams 2000). Hence, the correlation between body surface area to body mass ratio and aridity in the Common Bulbul is unlikely to be due to differences in net primary productivity, as frequently suggested for other animals (Yom-Tov and Geffen 2006, 2011). Geographical variation in body mass has previously been reported for the Common Bulbul (Crowe et al. 1981; Brittion 1972; Hanmer 1978): bulbuls tend to be heavier in localities with lower environmental temperatures and high productivity. These results, although from body mass records that were uncorrected for size in four bulbul subspecies with different geographic ranges, were interpreted as being consistent with Bergmann’s rule and the productivity hypothesis (Niles 1973). Our results do not negate these conclusions (Crowe et al. 1981), but we argue they may have been arrived at by chance because the occurrence of heavier birds (rather than birds with larger body surface area to volume ratio) in cooler environments does not necessarily imply conformity to Bergmann’s rule. Our raw body mass data also showed a negative non-significant trend with temperature (Fig. 2), but wing length, which is a comparatively better index of structural body size (Piersma and Davidson 1991), was significantly positively correlated with temperature (Fig. 2). We suggest that correlations of body surface area to body mass ratio with both temperature and aridity indicate a link between thermoregulation and water balance in dry environments (James 1970; Hudson and Bernstein 1981). Common Bulbuls had a smaller body surface area to mass ratio in more isothermal environments and larger body surface area to mass ratio in more seasonally arid environments (see negative correlations in Fig. 3 and positive correlations in Fig. 4, respectively), which suggests that relative body surface area may be adapted to both the effect of environmental temperature and aridity on internal water balance (Williams and Tieleman 2005). We propose that, besides several adjustments for thermoregulation (Tieleman and Williams 2000), birds may structurally adapt body size for non-evaporative heat loss so as to manage body temperature and reduce water loss in dry environments (Niles 1973).

Species with large distribution ranges or partial migratory status may show significant variation in body size, and this may correlate strongly with latitude (Yom-Tov and Geffen 2006), even if unrelated to Bergmann’s rule. We only confirm that the pattern observed in the locally resident Common Bulbul conforms to Bergmann’s rule after precluding any relationships between body mass and breeding with latitude, and confirming a significant positive correlation of wing length and latitude (Fig. 2). Therefore, we eliminate the possibility of misinterpreting an interrupted foraging response due to breeding (Nwaogu et al. 2017), starvation risk (Macleod and Gosler 2006) or migration (Hahn et al. 2015; Grilli et al. 2017) as conforming to Bergmann’s rule. Unfortunately, both wing length and body mass have been used on their own to test Bergmann’s rule (Watt et al. 2010) and this may lead to misleading interpretations. Bioclimatic variables explain 0–45 and 0–26% variation in wing length and body mass, respectively (Tables S1, S2), whereas they explain 0–63% variation in body surface area to mass ratio (Table 1). For birds, we suggest that squared wing length by body mass is a more informative proxy for relative body surface area than single measures of body size.

### Conclusion

Although Bergmann (and several more recent authors) used latitude and single linear measurements of body size to test conformity to Bergmann’s rule, its proposed mechanism (Salewski and Watt 2017) is independent of latitude and concerns body surface area to volume ratio, which requires a combination of area and cubic measurement of body size. It is thus likely that the validity of the mechanism proposed in Bergmann’s rule (Mayr 1956; Watt et al. 2010; Salewski and Watt 2017) has not actually been tested empirically on living animals—yet this is crucial for assessing its validity and applicability to tests for, and predicting the effects of global warming. The problem associated with testing Bergmann’s rule involves both data collection and utilisation, and our method may help with the former given the wealth of available data from bird-ringing schemes. Nonetheless, a combination of comparative morphometric analyses and translocation experiments may be used to further test the validity of Bergmann’s rule by exposing different populations that show body size conformity to Bergmann’s rule to controlled temperature conditions. In addition, by measuring indices such as heat stress, metabolic rates and heat/water loss, thermoregulatory differences arising from relative differences in body surface area may finally be proven.

## Electronic supplementary material

Below is the link to the electronic supplementary material.

Supplementary material 1 (DOCX 22 kb)
