# Supplementary material for: Temperature and aridity determine body size conformity to Bergmann’s rule independent of latitudinal differences in a tropical environment
Source: J Ornithol. 2018 Jun 27;159(4):1053–62. doi: 10.1007/s10336-018-1574-8 (PMC6417377; doi:10.1007/s10336-018-1574-8)
Supplement: Supplementary file 1 — Supplementary material 1 (DOCX 22 kb) [file 10336_2018_1574_MOESM1_ESM.docx]

**Supporting information**

**Temperature and aridity determine body size conformity to Bergmann’s rule independent of latitudinal differences in a tropical environment.**

Chima J. Nwaogu^1,2,3^, B. Irene Tieleman^1^, Kwanye Bitrus^3,4^ and Will Cresswell^2,3^

^1^Groningen Institute for Evolutionary Life Sciences, University of Groningen, P.O. Box 11103, 9700 CC, Groningen, The Netherlands.

^2^ School of Biology, University of St Andrews, Harold Mitchell Building, St Andrews Fife KY16 9TH, UK.

^3^ A.P. Leventis Ornithological Research Institute, Jos, Nigeria.

^4^ Department of Evolutionary Zoology and Human Biology, University of Debrecen, 4032, Debrecen, Egyetem tèr 1, Hungary.

***Correspondence:** [c.j.nwaogu@rug.nl](mailto:c.j.nwaogu@rug.nl)

**Table S1**: Adjusted R- squares of General Linear Models explaining wing length variation in Common bulbuls across environmental conditions in West Africa. Full model includes latitude, altitude and one bioclimatic variable as predictors. Change in adjusted R - squares is the difference that results after dropping the variable in a column heading from the full model with all three variables. Models where bioclimatic variable made significant contribution to explaining wing length are indicated in bold.

|  | **R^2^ of univariate model** | | |  | **Δ R^2^ after dropping variable from multivariate model** | | |
| --- | --- | --- | --- | --- | --- | --- | --- |
| Climatic Variable | Climatic variable | Latitude | Altitude | Full model | -Climate variable | -Latitude | -Altitude |
| Annual Mean Temperature | 0.008 | 0.3 | 0.11 | 0.39 | 0.01 | -0.07 | -0.02 |
| **Mean Diurnal Range (Mean of monthly (max temp - min temp))** | **0.4** | **0.3** | **0.11** | **0.42** | **-0.02** | **0.03** | **-0.05** |
| **Isothermality** | **0.24** | **0.3** | **0.11** | **0.44** | **-0.04** | **0.03** | **-0.17** |
| **Temperature Seasonality (standard deviation *100)** | **0.24** | **0.3** | **0.11** | **0.39** | **0.01** | **0.03** | **-0.12** |
| Max Temperature of warmest month | -0.0006 | 0.3 | 0.11 | 0.44 | -0.04 | 0.03 | -0.12 |
| **Min Temperature of coldest month** | **0.45** | **0.3** | **0.11** | **0.41** | **-0.01** | **0.03** | **0.03** |
| **Temperature annual range** | **0.36** | **0.3** | **0.11** | **0.44** | **-0.04** | **0.03** | **-0.11** |
| Mean Temperature of Wettest Quarter | -0.03 | 0.3 | 0.11 | 0.52 | -0.12 | 0.02 | -0.18 |
| **Mean Temperature of Driest Quarter** | **0.2** | **0.3** | **0.11** | **0.37** | **0.03** | **-0.16** | **0.02** |
| Mean Temperature of Warmest Quarter | -0.04 | 0.3 | 0.11 | 0.39 | 0.01 | 0.02 | -0.02 |
| Mean Temperature of Coldest Quarter | 0.14 | 0.3 | 0.11 | 0.37 | 0.03 | -0.28 | 0.01 |
| **Annual Precipitation** | **0.19** | **0.3** | **0.11** | **0.37** | **0.03** | **0** | **-0.1** |
| Precipitation of the Wettest Month | 0.008 | 0.3 | 0.11 | 0.37 | 0.03 | -0.14 | -0.09 |
| **Precipitation of the Driest Month** | **0.14** | **0.3** | **0.11** | **0.37** | **0.03** | **-0.12** | **-0.11** |
| **Precipitation Seasonality (Coefficient of Variation)** | **0.35** | **0.3** | **0.11** | **0.43** | **-0.03** | **0.02** | **-0.11** |
| Precipitation of the Wettest Quarter | 0.03 | 0.3 | 0.11 | 0.37 | 0.03 | -0.12 | -0.09 |
| **Precipitation of Driest Quarter** | **0.3** | **0.3** | **0.11** | **0.4** | **0** | **-0.01** | **-0.1** |
| **Precipitation of Warmest Quarter** | **0.18** | **0.3** | **0.11** | **0.45** | **-0.05** | **-0.01** | **-0.17** |
| Precipitation of Coldest Quarter | 0.08 | 0.3 | 0.11 | 0.39 | 0.01 | -0.16 | -0.08 |

**Table S2**: Adjusted R- squares of General Linear Models explaining body mass variation in Common bulbuls across environmental conditions in West Africa. Full model includes latitude, altitude and one bioclimatic variable as predictors. Change in adjusted R - squares is the difference that results after dropping the variable in a column heading from the full model with all three variables. Models where bioclimatic variable made significant contribution to explaining body mass are indicated in bold.

|  | **R2 of univariate model** | | |  | **Δ R2 after dropping variable from multivariate model** | | |
| --- | --- | --- | --- | --- | --- | --- | --- |
| Climatic Variable | Climatic variable | Latitude | Altitude | Full model | -Climatic variable | Latitude | Altitude |
| Annual Mean Temperature | 0.09 | 0.13 | 0.03 | 0.14 | 0.04 | -0.03 | 0.05 |
| Mean Diurnal Range (Mean of monthly (max temp - min temp)) | 0.09 | 0.13 | 0.03 | 0.14 | 0.04 | 0.04 | -0.05 |
| **Isothermality** | **0.26** | **0.13** | **0.03** | **0.21** | **-0.03** | **0.04** | **0.02** |
| **Temperature Seasonality (standard deviation *100)** | **0.16** | **0.13** | **0.03** | **0.14** | **0.04** | **0.04** | **-0.02** |
| **Max Temperature of warmest month** | **0.22** | **0.13** | **0.03** | **0.15** | **0.03** | **0.04** | **0.04** |
| Min Temperature of coldest month | -0.02 | 0.13 | 0.03 | 0.16 | 0.02 | 0.05 | -0.02 |
| **Temperature annual range** | **0.15** | **0.13** | **0.03** | **0.16** | **0.02** | **0.04** | **-0.05** |
| Mean Temperature of Wettest Quarter | 0.07 | 0.13 | 0.03 | 0.17 | 0.01 | -0.14 | -0.02 |
| Mean Temperature of Driest Quarter | -0.02 | 0.13 | 0.03 | 0.14 | 0.04 | -0.03 | 0.03 |
| **Mean Temperature of Warmest Quarter** | **0.19** | **0.13** | **0.03** | **0.17** | **0.01** | **0.05** | **0.04** |
| Mean Temperature of Coldest Quarter | 0.02 | 0.13 | 0.03 | 0.16 | 0.02 | -0.18 | 0.04 |
| **Annual Precipitation** | **0.26** | **0.13** | **0.03** | **0.24** | **-0.06** | **0.03** | **0.01** |
| **Precipitation of the Wettest Month** | **0.15** | **0.13** | **0.03** | **0.15** | **0.03** | **-0.03** | **0** |
| **Precipitation of the Driest Month** | **0.26** | **0.13** | **0.03** | **0.27** | **-0.09** | **0.04** | **-0.05** |
| **Precipitation Seasonality (Coefficient of Variation)** | **0.15** | **0.13** | **0.03** | **0.16** | **0.02** | **0.04** | **-0.06** |
| **Precipitation of the Wettest Quarter** | **0.16** | **0.13** | **0.03** | **0.15** | **0.03** | **-0.01** | **0** |
| **Precipitation of Driest Quarter** | **0.19** | **0.13** | **0.03** | **0.22** | **-0.04** | **-0.07** | **0.04** |
| **Precipitation of Warmest Quarter** | **0.22** | **0.13** | **0.03** | **0.19** | **-0.01** | **0.01** | **0** |
| **Precipitation of Coldest Quarter** | **0.17** | **0.13** | **0.03** | **0.16** | **0.02** | **0.03** | **-0.03** |

**Table S3**: Summary statistics of general linear model predicting body surface area to mass ratio of common bulbuls *Pycnonotus barbatus* in 22 locations on a temperature and aridity gradient in Nigeria. Body surface area was calculated as the square of wing length. Time and date of capture were not significant predictors of body surface area to mass ratio and were not included in the final model used to estimate predicted body surface area to mass ratio for analyses reported in table 1, and figures 1 and 2.

| Variable | Df | F | P |  |
| --- | --- | --- | --- | --- |
| Location | 21 | 13.34 | <0.001 | *** |
| Hour | 1 | 0.91 | 0.34 |  |
| Date | 1 | 1.98 | 0.16 |  |
